# Supplementary material for: Identification of and Mechanistic Insights into SARS-CoV-2 Main Protease Non-Covalent Inhibitors: An In-Silico Study
Source: Int J Mol Sci. 2023 Feb 20;24(4):4237. doi: 10.3390/ijms24044237 (PMC9959744; doi:10.3390/ijms24044237)
Supplement: Supplementary file 1 [file ijms-24-04237-s001.zip › Supplementary materials/Supplementary materials.pdf]

Identification of and Mechanistic Insights into SARS-CoV-2 Main Protease Non-Covalent Inhibitors: An In-Silico Study

Jian-Xin Shen[1,†], Wen-Wen Du[1,†], Yuan-Ling Xia[1], Zhi-Bi Zhang[1,2], Ze-Fen Yu[1], Yun-Xin Fu[1,3,*] and Shu-Qun Liu[1,*]

[1] State Key Laboratory for Conservation and Utilization of Bio-Resources in Yunnan & School of Life Sciences, Yunnan University, Kunming 650091, China; JianxinS0806@163.com (J.-X.S.); wenwendu2014@hotmail.com (W.-W.D.); xiayl@ynu.edu.cn (Y.-L.X.); zhangzhibi@kmmu.edu.cn (Z.-B.Z.); zfyu@ynu.edu.cn (Z.-F.Y.)

[2] Yunnan Key Laboratory of Stem Cell and Regenerative Medicine and Biomedical Engineering Research Center, Kunming Medical University, Kunming 650500, China

[3] Human Genetics Center and Department of Biostatistics and Data Science, School of Public Health, The University of Texas Health Science Center, Houston, TX 77030, USA

* Correspondence: Yunxin.Fu@uth.tmc.edu (Y.-X.F.); shuqunliu@ynu.edu.cn (S.-Q.L.)

† These authors contributed equally to this work

Table S1. ZINC IDs of the 593 hit compounds and their respective score values (kcal/mol) of the top-ranked docking pose (see the file Table_S1.xlsx).

Table S2. Predicted parameters of Lipinski's rule of five (Ro5) and ADMET for the 78 potential candidate compounds (PCCs) and the reference compound ML188 (see the file Table_S2.xlsx).

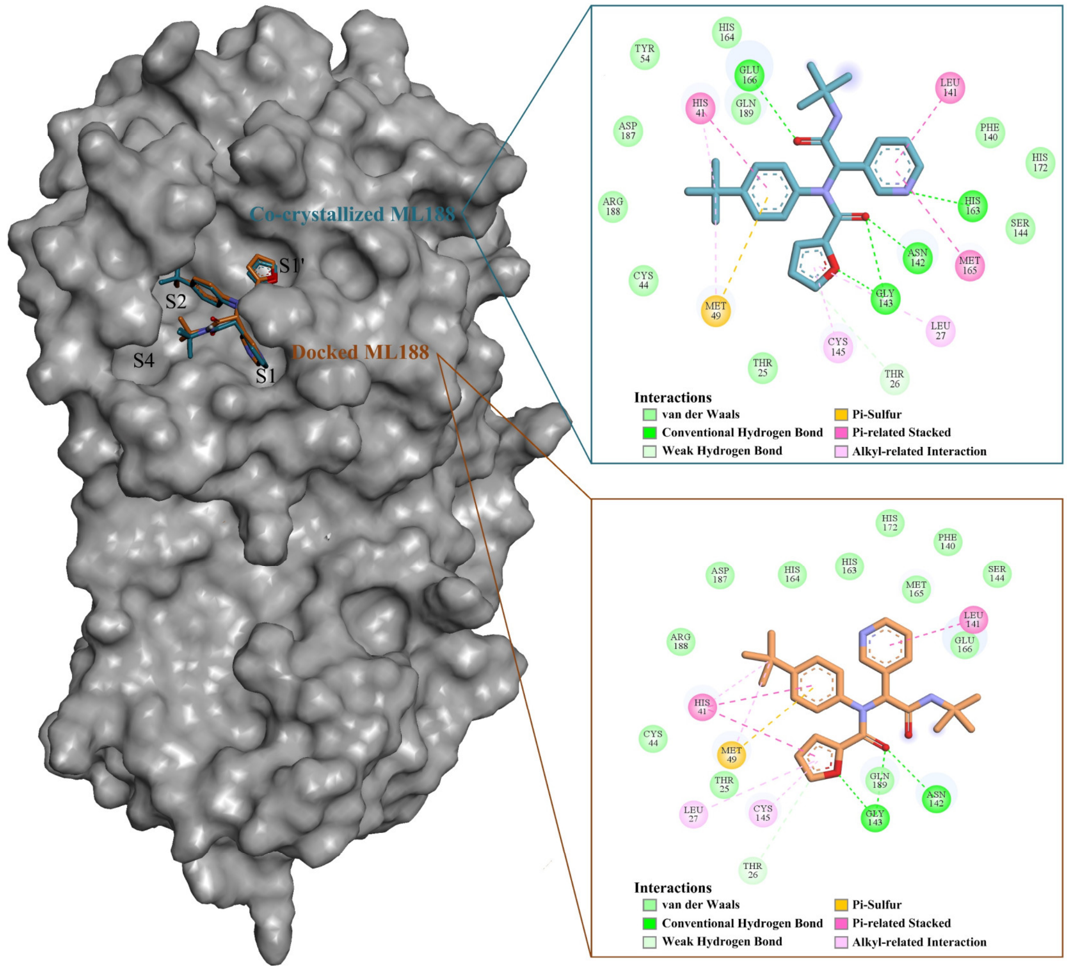

Figure S1. The co-crystallized and docked ML188 within the substrate-binding cavity of the SARS-CoV-2 Mpro. The two enlarged panels show the 2D representations of the binding modes of the co-crystallized ML188 (in dark cyan) and docked ML188 (in orange) with Mpro residues, respectively.

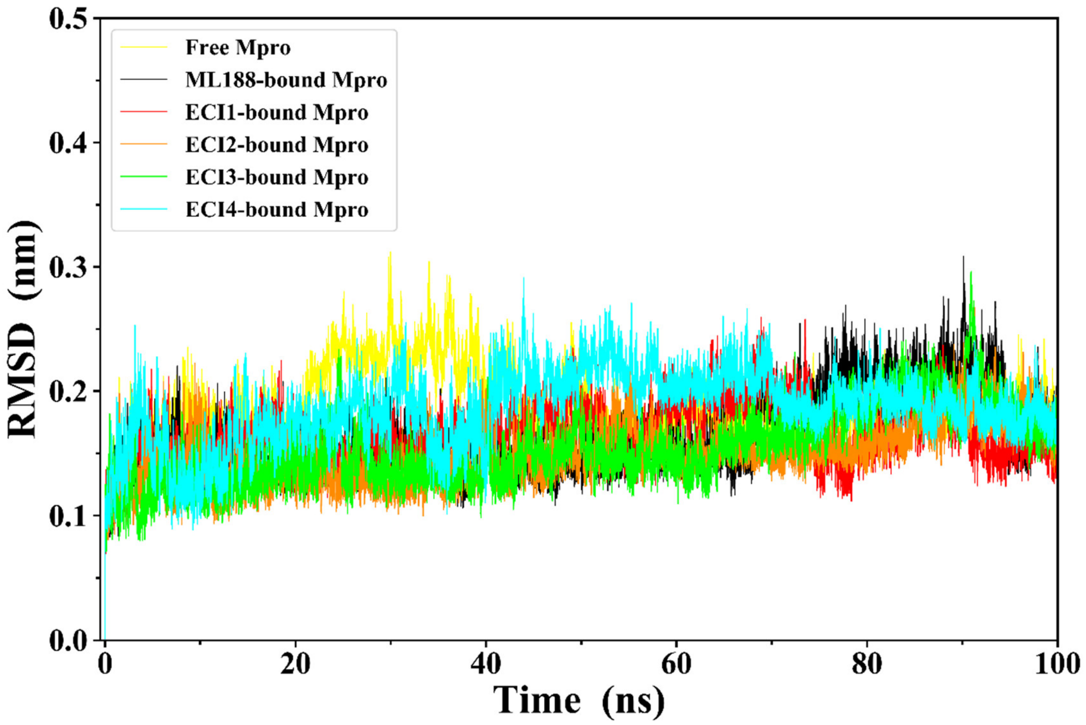

Figure S2. Time-dependent Cα root mean square deviation (RMSD) values of the free Mpro (without ligand) and the ligand-bound Mpro with respect to their respective starting structures during the 100-ns MD simulation.

File S1. Python script for constructing free energy landscapes (FELs) of the reference complex Mpro-ML188 and the three effective complexes Mpro-ECI2, Mpro-ECI3, and Mpro-ECI4 (see the file File_S1.py).
